# Supplementary material for: Complexome profiling of the Chlamydomonas psb28 mutant reveals TEF5 as an early PSII assembly factor
Source: Plant Cell. 2025 Mar 17;37(6):koaf055. doi: 10.1093/plcell/koaf055 (PMC12164590; doi:10.1093/plcell/koaf055)
Supplement: koaf055_Supplementary_Data [file koaf055_supplementary_data.zip › psb28_interactive.zip!/plots/intensity/qualityPlots/peptides/_Cre06.g278264.t1.1.html]

### Cre06.g278264.t1.1

Trivial name:   
SeqCoverage [median]: 9.50  
Mass: 39.30 kDa  
  
 

| Sequence | MissedCleavage | Mass [Da] | wtIntensity | muIntensity | Score |
| --- | --- | --- | --- | --- | --- |
| AIIGGSYGVSPTLVVR | 0 | 1587.90 | 726.54 | 4775.97 | 120.55 |
| EQQQADILR | 0 | 1099.56 | 0.00 | 1334.03 | 98.03 |
| QQFLDSVAELR | 0 | 1304.67 | 0.00 | 1991.86 | 87.91 |
|  |  |  | 726.5 | 8101.9 |  |
